# Determining external randomised pilot trial feasibility in preparation for a definitive trial: a web-based survey of corresponding authors of external pilot trial publications

**DOI:** 10.1186/s13063-022-06981-8

**Published:** 2023-01-24

**Authors:** Katie Mellor, Susan J. Dutton, Sally Hopewell

**Affiliations:** Oxford Clinical Trials Research Unit/Centre for Statistics in Medicine, Nuffield Department of Orthopaedics, Rheumatology and Musculoskeletal Sciences, University of Oxford, Oxford, England

**Keywords:** Pilot trials, Randomised controlled trials, Feasibility studies, Progression criteria

## Abstract

**Background:**

External randomised pilot trials aim to determine whether a future definitive randomised controlled trial (RCT) should be conducted, and if so, how. However, not every pilot trial that suggests that a definitive trial will be feasible will progress to a definitive study. In this study, we surveyed corresponding authors of external randomised pilot trial publications to assess pilot trial outcomes in terms of feasibility and progression.

**Methods:**

Web-based surveys were sent to corresponding authors of external randomised pilot trial publications, open for four weeks between January and February 2022. Four surveys were produced depending on whether the corresponding author had published a trial protocol or results publication, and whether progression criteria were reported. Surveys asked whether a future RCT was considered feasible, whether progression criteria were met (if applicable), what other factors informed the assessment of pilot trial feasibility, and whether the pilot trial has progressed to further research. Data was analysed using descriptive statistics and conventional content analysis.

**Results:**

98 of 276 corresponding authors completed the survey (average response rate of 36% across all surveys). Of these, 89 respondents indicated that their trial had completed. Ninety per cent of respondents who were corresponding authors of completed pilot trials stated that their pilot trial was either feasible (42/89, 47%) or feasible with changes to the trial design (38/89, 43%), yet only 66% (59/89) reported the intention to conduct a future definitive trial. Availability of funding for a future definitive trial and changing priorities of the Chief Investigator were the most common barriers to progression identified. Qualitative research findings was the most frequent factor considered both by corresponding authors who reported and who did not report progression criteria when determining trial feasibility.

**Conclusions:**

Just under one quarter (21/89, 24%) of respondents who considered their external randomised pilot trial to be feasible, or feasible with changes, did not intend to conduct a definitive trial highlighting research inefficiency and waste.

**Trial registration:**

Open Science Framework osf.io/d28hr [20 December 2021]

**Supplementary Information:**

The online version contains supplementary material available at 10.1186/s13063-022-06981-8.

## Background

Pilot trials are a type of feasibility study that aim to determine whether a future definitive randomised controlled trial (RCT) should be conducted, and if so, how [1]. External pilot trials are small stand-alone studies conducted before a definitive RCT, where any outcome data collected during the pilot trial does not contribute to a future definitive RCT analysis. This distinguishes them from internal pilot trials that are instead embedded within a definitive RCT often forming the first phase, where outcome data collected does contribute to the RCT analysis [2]. To determine whether the definitive RCT is feasible, researchers often prespecify progression criteria based on the pilot trials’ feasibility objectives, that guide interpretation of external pilot trial findings to decide whether and how the definitive RCT should be conducted [3, 4].

Qualitative research conducted by members of our research group highlighted that sometimes researchers are unable to obtain funding for further research despite demonstrating the feasibility of their external pilot trial [5]. This supports the findings of a previous review of external pilot trials funded by the National Institute for Health and care research (NIHR) Research for Patient Benefit (RfPB), which described that although a pilot or feasibility study might suggest that a definitive trial will be feasible this does not guarantee that funding applications for the main trial will be successful, or that the research team will pursue funding for the definitive study [6]. A previous analysis of external pilot trials published five or more years ago in six anaesthesia journals suggested that around half (54%) progressed onto definitive studies, despite all reporting the intention to progress to a future trial [7]. This lack of progression where a future study is deemed to be feasible has been identified as a source of research inefficiency and waste [6], yet barriers to external pilot trial progression from researchers perspectives have not been previously explored.

It has been suggested that authors of external pilot trials that do not report progression criteria or report unclear progression criteria, may be more optimistic in reporting that a definitive RCT is feasible [8], and previous research has highlighted lack of progression criteria reporting in external pilot trial protocols [9]. We previously conducted a methodological review to investigate the application and reporting of progression criteria in a more recent sample of the external randomised pilot trial protocol and results publications published in four major journals (*Pilot and Feasibility Studies*, *BMJ Open*, *Trials* and *PLoS One*) between January 2018 and December 2019 [10]. We also found that many external randomised pilot trial publications did not adequately report or propose prespecified progression criteria to inform whether to proceed to a future definitive randomised controlled trial.

We have since conducted an international web-based survey to explore the outcomes in terms of feasibility and progression of a recent cohort of external randomised pilot trials that we identified during our methodological review [10]. The primary objective was to examine how trial investigators assess external pilot trial feasibility both where trialists had and, had not, prespecified formal progression criteria in their pilot trial publications. The secondary objectives were to determine the number of external pilot trials that were considered feasible, the number that progressed to a definitive trial, how well progression criteria informed the assessment of trial feasibility from the trial investigators’ perspective, and to identify potential barriers to external pilot trial progression.

## Methods

### Protocol and registration

A protocol for this research is registered on the Open Science Framework: osf.io/d28hr [11]. Ethical approval was provided by the University of Oxford MS IDREC, reference R78375/RE001. This study is reported following the Consensus-Based Checklist for Reporting of Survey Studies (CROSS) [12].

### Study design

We conducted a web-based survey study that contained both closed and open-ended questions to collect quantitative and qualitative data.

### Sample characteristics

Trial investigators surveyed were corresponding authors of external pilot trial publications that were identified when conducting our earlier methodological review [10]. The sample of trial publications is reported in detail elsewhere [10], and the characteristics of included publications are presented in Supplementary file 1. To summarise, we searched four journals that are known to publish pilot trial protocol and results publications through PubMed for external pilot trial protocol and results publications between January 2018 and December 2019 inclusive: *BMJ Open*, *Pilot and Feasibility Studies*, *Trials* and *PLoS One*. The search terms used included ‘pilot’ or ‘feasibility’ in the title, and ‘trial’, ‘study’ or ‘protocol’ in the title or abstract [10]. We identified 160 external randomised pilot trial publications (37 completed trials and 123 protocols) that reported detailed progression criteria. We also identified 118 external randomised pilot trial publications (34 completed trials and 84 protocols) that did not report formal progression criteria but did report a recruitment or sample size target for their pilot trial, which have also been included in this study.

### Data collection

Data collection followed two distinct stages: a literature search and web-based survey of corresponding authors.

For the 207 external randomised pilot trial protocols identified in the previous methodological review (original sample in Table 1), one author (KM) searched PubMed in December 2021 by author name, trial name or acronym, and searched trial registries by trial registration number, to identify whether the external pilot trial result publication had been published since the methodological review was conducted. Any identified results publications were checked to see whether progression criteria were reported. We retrieved publicly available corresponding authors’ contact email for each included external pilot trial results publication, or the protocol publication where a corresponding results publication was not identified (forming the final sample in Table 1).

**Table 1:** Survey outlines included in the original sample (identified from previous methodological review) and final sample (following literature search for corresponding result publications)

Survey number	Progression criteria^a^	Publication type	Original sample	Final sample	Response rate
Survey 1	Yes	Protocol	123	55	19/55 (34%)
Survey 2	Yes	Results	37	108	41/108 (37%)
Survey 3	No	Protocol	84	41	8/41 (19%)
Survey 4	No	Results	34	72	30/72 (41%)
Total^b^	-	-	278	276	98/276 (36%)

^a^No: Did not report detailed progression criteria but did report prespecified targets relating to recruitment or sample size

^b^There were two instances where the protocol and results publications for the same external pilot trial were included in the original sample: since both relate to the same pilot trial, only the result publication was included in the final sample

We produced four web-based surveys using Jisc online surveys [13], detailed in Table 1. Corresponding authors were sent the survey version based on whether their publication reported the external pilot trial protocol or results, and whether progression criteria were prespecified. Surveys asked whether the external pilot trial found a future RCT to be feasible and whether the pilot trial has progressed to further research. Where publications reported progression criteria surveys asked whether they were met and whether any other factors informed the assessment of external pilot trial feasibility. Where publications did not report progression criteria surveys asked authors how they had determined the feasibility and what this was based on. The surveys for authors of external pilot trial protocols included additional questions at the start to determine whether the pilot trial had completed, and whether the findings had been published. Surveys did not collect any personal identifiable information about the respondent. Pdfs of all surveys have been published on the Open Science Framework (reference osf.io/7jnvx) [14].

### Survey development and administration

Two trial statisticians based at the Centre for Statistics in Medicine (CSM) piloted the surveys for authors of protocol publications and provided feedback on functionality and design. Only surveys one and three were piloted as they contained the same questions as surveys two and four but with additional questions at the start.

Personalised emails (including the corresponding author’s name, the title of their publication, and a unique survey URL link) were sent to each corresponding author by KM. Where automated email responses were received that provided an alternative email the email was forwarded on. Surveys were open from the 17th of January until the 14th of February 2022 and were designed to take between 5 and 10 min to complete. Non-respondents were sent a reminder email two weeks after the initial contact email, and all respondents were invited to enter a prize draw to win a £50 One4All voucher upon survey completion.

### Informed consent

An electronic information leaflet was provided as a pdf from the Jisc website. Study participants provided electronic consent before they were able to proceed to survey questions.

### Data analysis

Descriptive statistics were produced within Jisc to describe the external pilot trial findings with regards feasibility assessment and progression. KM used conventional content analysis [15] to analyse open-ended survey questions. Raw survey data was exported from Jisc into Excel files, which were then imported into NVivo (v12). KM inductively developed descriptive codes based on the data collected, using respondents’ own words where possible, and then counted the number of instances of coding units across different respondents to analyse the data [16]. KM grouped codes with similar or linked properties into categories following a descriptive rather than interpretive approach.

## Results

### Survey sample and response rates

The original sample of external pilot trial publications identified from the methodological review [10] included 278 randomised pilot trial publications (207 protocols and 71 results). There were two instances where the protocol and corresponding results publications for the same external pilot trial were included in the original sample, so only the results publication was included in the survey. Corresponding results publications were identified from the literature search for an additional 109 of the 207 protocols in the original sample. Five protocols that originally did not report progression criteria did in their results publication. The final sample of 276 publications, for which corresponding author email addresses were obtained, included 96 protocols and 180 result publications, see Table 1.

The survey response rate varied across the four surveys from 19 to 41%, also detailed in Table 1. In total, 98 of 276 trialists responded (average response rate of 36%).

### Responses from corresponding authors of protocol publications

In total, 27 (27/96, 28%) responses (survey 1 19/55; survey 3 8/41) were received from corresponding authors of protocol publications.

Most indicated that their external pilot trial was in the reporting or dissemination stage (18/27, 67%). Of these, five respondents indicated that they had published their external pilot trial findings, yet these five publications were not identified when searching the literature. The other 13 respondents indicated that they had not yet published their external pilot trial findings but intend to in the future. Nine respondents described that their external pilot trial was still in set up (2/27, 7%), conduct (4/27, 15%), or analysis (3/27, 11%) stage at the time of survey completion, and therefore were unable to complete all their respective surveys (survey 1 and survey 3).

### Responses from corresponding authors who reported progression criteria

In total, 55 respondents (55/163, 34%) completed all of surveys 1 and 2, see Table 2. Eighty-seven per cent of respondents stated that their external pilot trial was feasible (25/55, 45%) or was feasible with changes to the trial design (23/55, 42%). Only four respondents stated that their external pilot trial was not feasible (4/55, 7%) and three stated that feasibility was unclear (3/55, 5%). All three provided reasons for why they considered feasibility to be unclear including challenges with recruitment, COVID-19, intervention adherence, and implementing the intervention in the current context given the availability of funding.

**Table 2:** Survey responses from corresponding authors who reported progression criteria in their publication

		**Survey 1** **(*n*=19)**	
**Which of the following best describes the current pilot trial stage?**			
Trial planning and design		0 (0%)	
Set up		0 (0%)	
Conduct		3 (16%)	
Analysis		2 (11%)	
Reporting or dissemination		14 (74%)	
**Are the pilot trial findings published?**			
Yes		3 (21%)	
No		11 (79%)	
**Do you plan to publish the pilot trial findings in the future?**			
Yes		11 (100%)	
No		0 (0%)	
	**Total** **(*n*=55)**	**Survey 1** **(*n*=14**^a^**)**	**Survey 2** **(*n*=41)**
**What were the pilot trial findings in relation to the feasibility of a future definitive trial?**			
Future definitive trial is feasible	25 (45%)	4 (29%)	21 (51%)
Future definitive trial is feasible with changes to design^b^	23 (42%)	8 (57%)	15 (37%)
Future definitive trial is not feasible	4 (7%)	1 (7%)	3 (7%)
Feasibility of the future definitive trial is unclear^b^	3 (5%)	1 (7%)	2 (5%)
^b^**Do you intend to do any further feasibility assessment?**			
Yes	6 (23%)	1 (11%)	5 (29%)
No	20 (77%)	8 (89%)	12 (71%)
**To what extent was the prespecified progression criteria met?**			
Met all criteria	26 (47%)	5 (36%)	21 (51%)
Met some criteria	26 (47%)	8 (57%)	18 (44%)
Met none of the criteria	1 (2%)	1 (7%)	0 (0%)
Other	2 (4%)	0 (0%)	2 (5%)
**How helpful were progression criteria in informing the assessment of trial feasibility?**			
Very helpful	36 (65%)	10 (71%)	26 (63%)
Somewhat helpful	15 (27%)	3 (21%)	12 (29%)
Not helpful	1 (2%)	0 (0%)	1 (2%)
Not a consideration	3 (5%)	1 (7%)	2 (5%)
**Did other factors (in addition to progression criteria) inform the assessment of trial feasibility?**			
Yes	34 (62%)	8 (57%)	26 (63%)
No	21 (38%)	6 (43%)	15 (37%)
**Do you intend to conduct a future definitive trial?**			
Yes	37 (67%)	9 (64%)	28 (68%)
**Has funding for the definitive trial been applied for?**			
Yes	19 (51%)	3 (33%)	16 (57%)
Funding awarded	12 (63%)	2 (67%)	10 (63%)
Funding not awarded	4 (21%)	1 (33%)	3 (19%)
Application outcome unknown	3 (16%)	0 (0%)	3 (19%)
No	18 (49%)	6 (67%)	12 (43%)
**What best describes the current stage of the definitive trial?**			
Trial planning & design	25 (68%)	7 (78%)	18 (64%)
Set up	5 (14%)	1 (11%)	4 (14%)
Conduct	5 (14%)	1 (11%)	4 (14%)
Analysis	0 (0%)	0 (0%)	0 (0%)
Reporting/dissemination	1 (3%)	0 (0%)	1 (4%)
Did not answer	1 (3%)	0 (0%)	1 (4%)
No	15 (27%)	5 (36%)	10 (24%)
Did not answer	3 (5%)	0 (0%)	3 (7%)

Responses presented were reported by 55 trialists who reported progression criteria in their publication, and whose pilot trials had completed

^a^Although 19 respondents completed survey 1, only 14 described that their pilot trial was in the reporting or dissemination stage and so responded to the questions presented in this table

^b^Respondents were asked whether they would conduct further feasibility assessment only where they considered their pilot trial to be feasible with changes or where feasibility was unclear

Equal proportions of respondents described meeting all (26/55, 47%) or some (26/55, 47%) of their progression criteria. One respondent described meeting none of their progression criteria (1/55, 2%). Two respondents stated that they had not prespecified progression criteria; however, they had reported how they planned to assess the feasibility to decide whether to progress to a definitive RCT. The majority of respondents stated that progression criteria were very helpful in informing assessment of feasibility (36/55, 65%).

Over two thirds of respondents reported the intention to conduct a future definitive trial (37/55, 67%) with the majority stating that their intended definitive trial was in the trial planning and design stage (25/37, 68%). Fifteen respondents reported that they did not intend to do a future definitive trial (15/55, 27%) and three did not provide a response to this question (3/55, 5%). Just over half of those that stated the intention to do a future definitive trial had applied for further funding to do so (19/37, 51%). Of these, twelve had been awarded funding (12/19, 63%), four were unsuccessful (4/19, 21%) and three had not yet received their application outcome (3/19, 16%).

### Responses from corresponding authors who did not report progression criteria

In total, 34 respondents (34/113, 30%) completed all of surveys 3 and 4, see Table 3. Ninety-four per cent of respondents said that their external pilot trial was feasible (17/34, 50%) or was feasible with changes to the trial design (15/34, 44%). Only one responder said that their external pilot trial was not feasible (1/34, 3%) and one said that feasibility was unclear based on their external pilot trial findings (1/34, 3%), stating that although the trial and intervention could be delivered, there were local practical implementation issues around staffing and resource availability.

**Table 3:** Survey responses from corresponding authors who did not report progression criteria in their publication

		**Survey 3** **(*n*=8)**	
**Which of the following best describes the current pilot trial stage?**			
Trial planning and design		2 (25%)	
Set up		0 (0%)	
Conduct		1 (13%)	
Analysis		1 (13%)	
Reporting or dissemination		4 (50%)	
**Are the pilot trial findings published?**			
Yes		2 (50%)	
No		2 (50%)	
**Do you plan to publish the pilot trial findings in the future?**			
Yes		2 (100%)	
No		0 (0%)	
	**Total** **(*n*=34)**	**Survey 3** **(*n*=4**^a^**)**	**Survey 4** **(*n*=30)**
**What were the pilot trial findings in relation to the feasibility of a future definitive trial?**			
Future definitive trial is feasible	17 (50%)	2 (50%)	15 (50%)
Future definitive trial is feasible with changes to design^b^	15 (44%)	2 (50%)	13 (43%)
Future definitive trial is not feasible	1 (3%)	0 (0%)	1 (3%)
Feasibility of the future definitive trial is unclear^b^	1 (3%)	0 (0%)	1 (3%)
^b^**Do you intend to do any further feasibility assessment?**			
Yes	4 (25%)	0 (0%)	4 (29%)
No	12 (75%)	2 (100%)	10 (71%)
**Did you consider prespecifying progression criteria?**			
Yes	10 (29%)	1 (25%)	9 (30%)
No	23 (68%)	3 (75%)	20 (67%)
Did not answer	1 (3%)	0 (0%)	1 (3%)
**Do you intend to conduct a future definitive trial?**			
Yes	22 (65%)	2 (50%)	20 (67%)
**Has funding for the definitive trial been applied for?**			
Yes	15 (68%)	0 (0%)	15 (75%)
Funding awarded	9 (60%)	0 (0%)	9 (60%)
Funding not awarded	4 (27%)	0 (0%)	4 (27%)
Application outcome unknown	2 (13%)	0 (0%)	2 (13%)
No	7 (32%)	2 (100%)	5 (25%)
**What best describes the current stage of the definitive trial?**			
Trial planning and design	12 (55%)	2 (100%)	10 (50%)
Set up	3 (14%)	0 (0%)	3 (15%)
Conduct	3 (14%)	0 (0%)	3 (15%)
Analysis	2 (10%)	0 (0%)	2 (10%)
Reporting/dissemination	1 (5%)	0 (0%)	1 (5%)
Did not answer	1 (5%)	0 (0%)	1 (5%)
No	12 (35%)	2 (50%)	10 (33%)

Responses presented were reported by 34 trialists who did not report progression criteria in their publication, and whose pilot trials had completed

^a^Although 8 respondents completed survey 3, only 4 described that their pilot trial was in the reporting or dissemination stage and so responded to the questions presented

^b^Respondents were asked whether they would conduct further feasibility assessment where they considered their pilot trial to be feasible with changes or where feasibility was unclear

Over two thirds of respondents did not consider prespecifying progression criteria for their external pilot trial (23/34, 68%). Ten respondents described having considered prespecifying progression criteria (10/34, 29%), of these, five stated that they had done so. However, we were unable to identify explicit criteria against which feasibility would be assessed within the publication.

Around two thirds of respondents reported the intention to conduct a future definitive trial (22/34, 65%) with the majority also stating that their intended definitive trial was in the trial planning and design stage (12/22, 55%). Over two thirds of these respondents described having applied for further funding (15/22, 65%): nine were awarded funding (9/15, 60%), four were unsuccessful (4/15, 27%) and two had not yet received their application outcome (2/15, 13%). Twelve respondents reported that they did not intend to conduct a future definitive trial (12/34, 35%).

### Proposed changes to the future definitive trial

Survey branching logic was used to invite participants who indicated that a future definitive trial would be feasible with changes to design (38/98, 39% of respondents across all four surveys) to specify what changes they would make. This information was offered by 37 of the 38 respondents, who often described the intention to make more than one change.

The most frequent change related to the intervention (17/37, 46%) with nine respondents describing how their intervention needed further development or refinement, eight suggesting that they would change their mode of intervention delivery, and two describing how more training would be provided to those delivering the intervention. Twelve respondents suggested that they would make changes to their recruitment strategy (12/37, 32%) including recruiting more sites, recruiting sites simultaneously rather than sequentially and altering the recruitment timeframe. Changes were also often described in relation to trial outcome measures (5/37, 14%), methods of data collection or follow-up (5/37, 14%), the patient population or eligibility criteria (4/37, 11%) and the overall trial design (4/37, 11%; e.g. adding or dropping arms, and moving to a cluster randomised design).

### Other considerations for external pilot trial progression

Over 60% of respondents who had authored external pilot trial publications reported progression criteria (surveys 1 and 2) described considering other factors when determining trial feasibility (34/55, 62%). All but one (33/34, 97%) provided further details about other factors considered, see Table 4.

**Table 4:** Specified factors that inform assessment of trial feasibility in addition to, or instead of, progression criteria

**Specified factors by corresponding authors of publications that reported progression criteria**^a^	
	**(*n*=33)**
Qualitative data	12 (36%)
Process evaluation	5 (15%)
Participant feedback^b^	3 (9%)
Qualitative interview data	3 (9%)
Qualitative data about implementation	1 (3%)
Trial design	6 (18%)
Outcome measures	3 (9%)
Data collection	1 (3%)
Performance of trial pathways	1 (3%)
Protocol adherence	1 (3%)
Selection bias	1 (3%)
Recruitment	5 (15%)
Recruitment processes and ability to recruit	4 (12%)
Difficulty with screening	1 (3%)
Contextual challenges	4 (12%)
COVID-19	2 (6%)
Healthcare context	2 (6%)
Changing policy	1 (3%)
Implementation of the trial	3 (9%)
Resources required	2 (6%)
Enthusiasm of researchers	1 (3%)
Number of recruiting sites needed	1 (3%)
Funding considerations	3 (9%)
Indication of efficacy or effectiveness	3 (9%)
Interest, acceptability or uptake of intervention	3 (9%)
Expectations of collaborators	1 (3%)
Retention or attrition	1 (3%)
**Specified factors by corresponding authors of publications that did not report progression criteria**^c^	
	**(*n*=32)**
Qualitative data	17 (53%)
Acceptability to participants^b^	11 (34%)
Participant feedback^b^	3 (9%)
Qualitative data about implementation	3 (9%)
Qualitative interview data	2 (6%)
Acceptability to healthcare providers^b^	2 (6%)
Process evaluation	1 (3%)
Recruitment	14 (44%)
Recruitment rate	11 (34%)
Recruitment processes	2 (6%)
Consent rate	1 (3%)
Recruitment time	1 (3%)
Indication of efficacy or effectiveness	10 (31%)
Trial design	10 (31%)
Sample size required for the definitive RCT	5 (16%)
Data collection	3 (9%)
Ability to do internal pilot as part of future def RCT	1 (3%)
Need to further development the intervention (not possible in an RCT)	1 (3%)
Retention or attrition	9 (28%)
Interest, acceptability or uptake of intervention	9 (28%)
Intervention adherence or engagement	5 (16%)
Completion or withdrawal rates	3 (9%)
Willingness to be randomised	1 (3%)
Implementation of the intervention	7 (22%)
Intervention delivery	3 (9%)
Intervention fidelity	2 (6%)
Intervention feasibility	1 (3%)
Complexity of the intervention	1 (3%)
Implementation of the trial	4 (13%)
Acceptability or willingness of healthcare professionals	2 (6%)
Patient acceptability of study procedures	1 (3%)
Resources required	1 (3%)
Safety or adverse events	3 (9%)
Contextual challenges	1 (3%)
Healthcare context	1 (3%)
Funding considerations	1 (3%)

Most participants mentioned more than one factor

^a^Responses presented were reported by 33 of 34 trialists who considered other factors, in addition to their progression criteria, to assess trial feasibility

^b^Unclear whether participant feedback and acceptability data were collected through formal qualitative research methods

^c^Responses presented were reported by 32 of 34 trialists who authored pilot trial publications that did not include progression criteria

The most frequent consideration was qualitative research findings (12/33, 36%), including process evaluations and qualitative data about implementation. Considerations about trial design, such as choice of outcome measures, methods of data collection, ability to reduce selection bias, performance of trial pathways and protocol adherence informed assessment of trial feasibility for six respondents (6/33, 18%). Five respondents stated that recruitment and screening processes and challenges (5/33, 15%), and four stated that contextual challenges (4/33, 12%), such as the impact of COVID-19, changes to the healthcare context and policies, informed assessment of trial feasibility. Implementation of the trial (including the resources and number of sites required, and researcher enthusiasm) informed feasibility assessment for three respondents (3/33, 9%).

### Findings that informed feasibility assessment where progression criteria were not stipulated

Of the 34 respondents who had authored external pilot trial publications that did not include progression criteria (surveys 3 and 4), 32 (32/34, 94%) provided details about the findings they considered when determining feasibility, see Table 4.

The most frequent consideration was also qualitative research findings (17/32, 53%). Fourteen respondents described that recruitment had informed their assessment of trial feasibility (14/32, 44%), such as considering the recruitment rate, recruitment processes, consent rate, and timeframe associated with recruitment. Ten respondents considered whether there was any indication of efficacy or effectiveness when deciding whether a definitive trial was feasible (10/32, 31%). Ten respondents described considering findings in relation to the definitive trial design (10/32, 31%). For example, whether the sample size required for the definitive trial was achievable and the methods of data collection. Nine respondents described considering retention or attrition when determining feasibility (9/32, 28%), and nine described considering whether there was sufficient interest, acceptability or uptake of intervention (9/32, 28%) including intervention adherence or engagement, completion or withdrawal rates, and willingness to be randomised. Seven respondents described considering whether the intervention could be implemented (7/32, 22%; for example deliverability and intervention fidelity), and four described considering implementation of the trial (4/32, 13%; for example, resources and sites required, willingness of healthcare providers to implement the study and acceptability of study procedures).

### Identified barriers to progression of a feasible external pilot trial to a future trial

Of the 80 respondents across all four surveys who stated that their future trial would be feasible or feasible with changes to the trial design (80/89, 90%), 18 (18/80, 23%) indicated that they did not intend to conduct a future definitive trial. Using survey branching logic these respondents were invited to provide reasons for why they did not intend to conduct a definitive trial (offered by 17/18, 94%), summarised in Table 5, with many providing more than one reason.

**Table 5:** Reported barriers to progression of feasible external pilot trials

Identified barrier	(***n***=17) ***n*** (%)
Funding considerations	6 (35%)
Chief Investigator priorities	5 (29%)
Healthcare context	3 (18%)
Changing healthcare landscape	1 (6%)
COVID-19	1 (6%)
Intervention is no longer required	1 (6%)
Indication of efficacy or effectiveness	3 (18%)
No indication of efficacy or effectiveness	2 (12%)
A different trial has provided evidence of efficacy	1 (6%)
Resources required	2 (12%)
Definitive trial sample size required is too large	1 (6%)
Further feasibility or piloting is required	1 (6%)
Lack of participant interest in the intervention	1 (6%)
Recruitment was difficult	1 (6%)
Reliability of data	1 (6%)
Success of collaborations	1 (6%)

Responses presented were reported by 17 respondents who considered their external pilot trial to be feasible or feasible with changes, but did not intend to conduct a future definitive trial

Most participants provided more than one reason

Funding considerations, such as whether funding for the definitive trial would be available and sufficient, were the most frequently reported reason for not doing a definitive trial, reported by six respondents (6/17, 35%). Changing Chief Investigator (CI) priorities was another more frequent barrier to external pilot trial progression (5/17, 29%): five respondents reported that they had decided to pursue other research interests instead of a definitive trial, with two stating that their external pilot trial had formed part of their PhD, and that they were not resourced to take it forward. Three respondents (3/17, 18%) described how changes to the healthcare context e.g. service delivery, implementation of other interventions, or the impact of COVID-19 meant that they could not justify a definitive trial. Two respondents described that they would not pursue a definitive trial because there was no indication of efficacy, and one described how efficacy had since been proven in a different fully powered RCT (2/17, 12%). Other barriers included the number of resources required (2/17, 12%), the definitive trial sample size was too large (1/17, 6%), further feasibility assessment was needed (1/17, 6%), a lack of interest in the intervention (1/17, 6%), challenges with recruitment (1/17, 6%), reliability of data collection (1/17, 6%) and unsuccessful stakeholder collaborations (1/17, 6%).

## Discussion

### Summary of main findings

Ninety per cent of the 89 respondents across all four surveys whose external pilot trial had completed stated that their pilot trial was either feasible (42/89, 47%) or feasible with changes to the trial design (38/89, 43%), yet only two-thirds reported the intention to conduct a future definitive trial (59/89, 66%). This suggests that just under one quarter (21/89, 24%) of respondents who considered their external pilot trial to be feasible (or feasible with changes) did not intend to conduct a definitive trial. Respondents described varied barriers to external pilot trial progression, with the availability of funding for a future definitive trial and changing priorities of the CI most common.

### Strengths and weaknesses of the study

A study strength is our comprehensive use of open and closed questions to appropriately address the study aim and objectives, with open-ended questions allowing respondents to expand on answers provided. However, only one researcher conducted the content analysis of open-ended questions which might have led to coding errors.

Although our response rate (average 36%) could be considered low, it was on par with response rates observed in other studies where trialists have been surveyed [17, 18]. Interestingly, our response rate amongst authors of external pilot trial protocols was lower than authors of external pilot trial results publications. This finding might be explained by publication bias, with non-feasible pilot trial result publications perhaps less likely to be published [8], and authors more willing to complete the survey where external pilot trials were feasible, had completed and been published. The COVID-19 pandemic likely also impacted and delayed many of the pilot trials included in this study and many might not have proceeded to completion in the timeframe between protocol publication and survey administration. In addition, all questions were optional rather than required, and some respondents chose not to respond to all questions.

A further limitation was that our email was not delivered to all identified corresponding authors for example where researchers were on leave or had moved to a new role as suggested by automated email responses. This meant that some identified corresponding authors did not get the opportunity to participate.

### Meaning of the study: possible explanations and implications for clinicians and policymakers

It has been suggested that authors of external pilot trials that do not report progression criteria or report unclear progression criteria, may be optimistic in reporting that a definitive RCT is feasible [8]. However, our findings indicated only slightly more respondents who did not report clear progression criteria in their external pilot trial publication considered their pilot trial to be feasible or feasible with changes compared to those who did include progression criteria (94%, 32/34 versus 87%, 48/55 respectively). This could indicate that even where progression criteria are reported researchers might be over optimistic in reporting that their external pilot trial is feasible.

The feasibility rates observed in this study were higher than those that have been previously reported in external pilot trials, ending between 1995 and 2019, that were registered on the International Standard Randomised Controlled Trials Number (ISRCTN) registry (83%) [8], and in NIHR RfPB funded external pilot trials that had completed by May 2016 (64%) [6]. Since the sample of external pilot trials included in this review was more recent, these findings might suggest that the proportion of pilot trials that indicate feasibility has increased. However, it is also possible that authors of external pilot trials that were feasible were simply more likely to respond to the survey.

It has also been previously suggested that some external pilot trials might be redundant, i.e. they are conducted without sufficient uncertainty about feasibility that they are likely to show that an RCT will be feasible, and so waste time and resources [8]. Our findings support this hypothesis and suggest that some external pilot trials might be contributing to research waste if they are conducted with no intention to progress to further research.

### Unanswered questions and future research

Our findings suggest that more guidance is needed to ensure that external pilot trial protocol and results publications can be identified from the literature. Five respondents who were sent the appropriate survey for corresponding authors of external pilot trial protocols indicated that they had published their pilot trial results, yet we did not identify these publications. This highlights the difficulty in linking external pilot trial protocols to results publications, even when a systematic approach is used. Possible explanations for this are that the corresponding first author changed, that the external pilot trial title or acronym changed, or that the results publication was published in the time between the literature search and sending of the survey.

Our findings also highlight the need for clearer guidance for progression criteria reporting. Some authors did not necessarily agree with our assessment of progression criteria reporting. Potential explanations for this are that authors had prespecified progression criteria, but this was not clearly reported in the publication, or that interpretations of progression criteria differ, which is perhaps likely given that there is no clear guidance for progression criteria. To address this, research is ongoing to develop guidance specific to the application and reporting of progression criteria in external randomised pilot trials.

## Conclusions

We found that although 90% of respondents across all surveys whose trial had completed considered their external pilot trial to be either feasible or feasible with changes, only two-thirds reported the intention to conduct a future definitive trial, indicating potential inefficiency and research waste. Availability of funding and changing CI priorities were the two most frequent barriers to progression identified.

## Supplementary Information

**Additional file 1.** Characteristics of included publications. Reporting guidelines: CROSS reporting checklist.

## Data Availability

Data will be made available upon request and included in a DPhil thesis published open access through the Oxford University Archive upon KM’s DPhil completion.

## Abbreviations

CI: Chief Investigator
CROSS: Consensus-Based Checklist for Reporting of Survey Studies
CSM: Centre for Statistics in Medicine
ISRCTN: International Standard Randomised Controlled Trials Number
MS IDREC: Medical Sciences Interdivisional Research Ethics Committee
NIHR: National Institute for Health and care research
RCT: Randomised Controlled Trial
RfPB: Research for Patient Benefit
TSC: Trial Steering Committee
