# Supplementary material for: Determining external randomised pilot trial feasibility in preparation for a definitive trial: a web-based survey of corresponding authors of external pilot trial publications
Source: Trials. 2023 Jan 24;24:53. doi: 10.1186/s13063-022-06981-8 (PMC9871417; doi:10.1186/s13063-022-06981-8)
Supplement: Supplementary file 1 — Additional file 1. Characteristics of included publications. Reporting guidelines: CROSS reporting checklist. [file 13063_2022_6981_MOESM1_ESM.docx]

|  | **Report progression criteria** | | | **Comparator subset*** | | |
| --- | --- | --- | --- | --- | --- | --- |
|  | **Total  (n = 160)** | **Completed (n = 37)** | **Protocol (n = 123)** | **Total (n = 118)** | **Completed (n = 34)** | **Protocol (n = 84)** |
|  | **n (%)** | **n (%)** | **n (%)** | **n (%)** | **n (%)** | **n (%)** |
| **Journal** |  |  |  |  |  |  |
| *British Medical Journal (BMJ) Open* | 45 (28%) | 11 (30%) | 34 (28%) | 46 (39%) | 11 (31%) | 35 (42%) |
| *Pilot and Feasibility Studies (PAFS)* | 77 (48%) | 21 (57%) | 56 (46%) | 36 (31%) | 16 (46%) | 20 (24%) |
| *Trials* | 35 (22%) | 2 (5%) | 33 (27%) | 35 (30%) | 6 (18%) | 29 (35%) |
| *Public Library of Science (PLoS) One* | 3 (2%) | 3 (8%) | 0 (0%) | 1 (1%) | 1 (1%) | 0 (0%) |
|  |  |  |  |  |  |  |
| **Country** |  |  |  |  |  |  |
| Australia | 10 (6%) | 6 (16%) | 4 (3%) | 13 (11%) | 3 (9%) | 10 (12%) |
| Brazil | 1 (1%) | 0 (0%) | 1 (1%) | 1 (1%) | 0 (0%) | 1 (1%) |
| Canada | 19 (12%) | 4 (11%) | 15 (12%) | 4 (3%) | 0 (0%) | 4 (5%) |
| Chile | 0 (0%) | 0 (0%) | 0 (0%) | 2 (2%) | 0 (0%) | 2 (2%) |
| China | 4 (3%) | 0 (0%) | 4 (3%) | 2 (2%) | 0 (0%) | 2 (2%) |
| Denmark | 1 (1%) | 0 (0%) | 1 (1%) | 2 (2%) | 0 (0%) | 2 (2%) |
| Germany | 2 (1%) | 1 (3%) | 1 (1%) | 5 (4%) | 2 (6%) | 3 (4%) |
| Indonesia | 0 (0%) | 0 (0%) | 0 (0%) | 1 (1%) | 0 (0%) | 1 (1%) |
| Italy | 0 (0%) | 0 (0%) | 0 (0%) | 3 (3%) | 0 (0%) | 3 (4%) |
| Korea | 1 (1%) | 0 (0%) | 1 (1%) | 1 (1%) | 0 (0%) | 1 (1%) |
| Nepal | 3 (2%) | 1 (3%) | 2 (2%) | 0 (0%) | 0 (0%) | 0 (0%) |
| New Zealand | 3 (2%) | 2 (5%) | 1 (1%) | 1 (1%) | 1 (3%) | 0 (0%) |
| Norway | 1 (1%) | 1 (3%) | 0 (0%) | 3 (3%) | 1 (3%) | 2 (2%) |
| Republic of Ireland | 5 (3%) | 0 (0%) | 5 (4%) | 1 (1%) | 0 (0%) | 1 (1%) |
| Sri Lanka | 0 (0%) | 0 (0%) | 0 (0%) | 1 (1%) | 0 (0%) | 1 (1%) |
| Sweden | 2 (1%) | 1 (3%) | 1 (1%) | 1 (1%) | 1 (3%) | 0 (0%) |
| Switzerland | 0 (0%) | 0 (0%) | 0 (0%) | 2 (2%) | 1 (3%) | 1 (1%) |
| Tanzania | 1 (1%) | 0 (0%) | 1 (1%) | 0 (0%) | 0 (0%) | 0 (0%) |
| Thailand | 1 (1%) | 0 (0%) | 1 (1%) | 0 (0%) | 0 (0%) | 0 (0%) |
| The Netherlands | 2 (1%) | 0 (0%) | 2 (2%) | 0 (0%) | 0 (0%) | 0 (0%) |
| Uganda | 0 (0%) | 0 (0%) | 0 (0%) | 1 (1%) | 0 (0%) | 1 (1%) |
| UK | 87 (54%) | 19 (51%) | 68 (55%) | 66 (56%) | 21 (62%) | 45 (54%) |
| USA | 16 (10%) | 2 (5%) | 14 (11%) | 8 (7%) | 4 (12%) | 4 (5%) |
| Zimbabwe | 1 (1%) | 0 (0%) | 1 (1%) | 0 (0%) | 0 (0%) | 0 (0%) |
|  |  |  |  |  |  |  |
| **Funder** |  |  |  |  |  |  |
| Industry | 4 (3%) | 2 (5%) | 2 (2%) | 1 (1%) | 0 (0%) | 1 (1%) |
| Non-industry | 147 (92%) | 32 (86%) | 115 (94%) | 108 (92%) | 31 (91%) | 77 (92%) |
| A combination | 5 (3%) | 1 (3%) | 4 (3%) | 6 (5%) | 1 (3%) | 5 (6%) |
| Unknown | 3 (2%) | 2 (5%) | 1 (1%) | 3 (3%) | 2 (6%) | 1 (1%) |
| Trial did not receive funding | 1 (1%) | 0 (0%) | 1 (1%) | 0 (0%) | 0 (0%) | 0 (0%) |
|  |  |  |  |  |  |  |
| **Therapeutic area**** |  |  |  |  |  |  |
| Psychiatry/Psychology | 21 (13%) | 2 (5%) | 19 (15%) | 18 (15%) | 0 (0%) | 18 (21%) |
| Public Health | 17 (11%) | 2 (5%) | 15 (12%) | 11 (9%) | 6 (18%) | 5 (6%) |
| Neurology | 15 (9%) | 3 (8%) | 12 (10%) | 13 (11%) | 6 (18%) | 7 (8%) |
| Oncology | 11 (7%) | 4 (11%) | 7 (6%) | 12 (10%) | 1 (3%) | 11 (13%) |
| Surgery | 11 (7%) | 3 (8%) | 8 (7%) | 4 (3%) | 2 (6%) | 2 (2%) |
| Musculoskeletal | 10 (6%) | 6 (16%) | 4 (3%) | 2 (2%) | 2 (6%) | 0 (0%) |
| Trauma | 9 (6%) | 2 (5%) | 7 (6%) | 4 (3%) | 1 (3%) | 3 (4%) |
| Critical Care | 8 (5%) | 1 (3%) | 7 (6%) | 0 (0%) | 0 (0%) | 0 (0%) |
| Obstetrics/Gynaecology | 8 (5%) | 2 (5%) | 6 (5%) | 4 (3%) | 1 (3%) | 3 (4%) |
| Endocrinology | 6 (4%) | 0 (0%) | 6 (5%) | 2 (2%) | 2 (6%) | 0 (0%) |
| Geriatrics | 5 (3%) | 1 (3%) | 4 (3%) | 10 (8%) | 3 (9%) | 7 (8%) |
| Paediatrics | 5 (3%) | 2 (5%) | 3 (2%) | 4 (3%) | 1 (3%) | 3 (4%) |
| Other | 34 (21%) | 9 (24%) | 25 (20%) | 34 (29%) | 9 (26%) | 25 (30%) |
|  |  |  |  |  |  |  |
| **Intervention type** |  |  |  |  |  |  |
| Drug | 13 (8%) | 4 (11%) | 9 (7%) | 11 (9%) | 1 (3%) | 10 (12%) |
| Surgery/procedure | 19 (12%) | 6 (16%) | 13 (11%) | 7 (5%) | 3 (9%) | 4 (5%) |
| Counselling/lifestyle/physiotherapy | 94 (59%) | 22 (59%) | 72 (59%) | 59 (50%) | 18 (53%) | 41 (49%) |
| Equipment | 9 (6%) | 4 (11%) | 5 (4%) | 9 (7%) | 3 (9%) | 6 (7%) |
| Other | 25 (16%) | 1 (3%) | 24 (20%) | 32 (27%) | 9 (26%) | 23 (27%) |
|  |  |  |  |  |  |  |
| **Sample size target***** |  |  |  |  |  |  |
| Mean (SD) | 217.3 (1074.9) | 72.8 (62.5) | 258.5 (1215.7) | 87.5 (90.6) | 64 (51.8) | 97.0 (101.0) |
| Median (IQR) | 60 (40-100) | 60 (32-90) | 60 (40-100) | 60 (40-100) | 60 (30-80) | 62 (44-116) |
| Min-Max | 6-12000 | 6-300 | 20-12000 | 10-700 | 10-300 | 12-700 |
| **Cluster randomised pilot trials** | **(n=21)** | **(n=3)** | **(n=18)** | **(n=14)** | **(n=7)** | **(n=7)** |
| Mean number of clusters | 9.3 (10.7) | 7.3 (2.3) | 9.7 (11.6) | 6.2 (3.8) | 6.9 (4.6) | 5.6 (2.9) |
| Median (IQR) | 6 (4-10) | 6 (6-10) | 6 (3-10) | 6 (4-8) | 6 (4-8) | 6 (2-8) |
| Min-Max | 2-45 | 6-10 | 2-45 | 2-16 | 2-16 | 2-9 |
|  |  |  |  |  |  |  |
| **Number of arms** |  |  |  |  |  |  |
| 2 | 143 (89%) | 32 (86%) | 111 (90%) | 102 (86%) | 28 (82%) | 74 (88%) |
| >2 | 17 (11%) | 5 (14%) | 12 (10%) | 16 (14%) | 6 (18%) | 10 (12%) |
|  |  |  |  |  |  |  |
| **Number of centres** |  |  |  |  |  |  |
| Single centre | 55 (34%) | 19 (51%) | 36 (29%) | 40 (34%) | 13 (38%) | 27 (32%) |
| Multi-centre | 102 (64%) | 18 (49%) | 84 (68%) | 76 (64%) | 20 (59%) | 56 (67%) |
| Unclear | 3 (2%) | 0 (0%) | 3 (2%) | 2 (2%) | 1 (3%) | 1 (1%) |
|  |  |  |  |  |  |  |
| **Feasibility objective/s explicitly described as primary** |  |  |  |  |  |  |
| Yes | 71 (44%) | 9 (24%) | 62 (50%) | 41 (35%) | 8 (24%) | 33 (39%) |
| No | 89 (56%) | 28 (76%) | 61 (50%) | 77 (65%) | 26 (76%) | 51 (61%) |
|  |  |  |  |  |  |  |
| **Trial outcomes address trial objectives** |  |  |  |  |  |  |
| Yes | 109 (68%) | 18 (49%) | 91 (74%) | 61 (52%) | 17 (50%) | 44 (52%) |
| No | 1 (1%) | 0 (0%) | 1 (1%) | 2 (2%) | 0 (0%) | 2 (2%) |
| Somewhat**** | 50 (31%) | 19 (51%) | 31 (25%) | 55 (47%) | 17 (50%) | 38 (45%) |
|  |  |  |  |  |  |  |
| **Hypothesis testing** |  |  |  |  |  |  |
| Yes | 18 (11%) | 2 (5%) | 16 (13%) | 16 (14%) | 2 (6%) | 14 (17%) |
| Yes, exploratory/caution advised | 61 (38%) | 18 (49%) | 43 (35%) | 28 (24%) | 12 (35%) | 16 (19%) |
| No | 81 (51%) | 17 (46%) | 64 (52%) | 74 (63%) | 20 (59%) | 54 (64%) |
|  |  |  |  |  |  |  |
| **Number of uncertainties reported** |  |  |  |  |  |  |
| One | 1 (1%) | 0 (0%) | 1 (1%) | 0 (0%) | 0 (0%) | 0 (0%) |
| Multiple | 159 (99%) | 37 (100%) | 122 (99%) | 118 (100%) | 34 (100%) | 84 (100%) |
|  |  |  |  |  |  |  |
| **Where uncertainties are first reported (excluding abstract)** |  |  |  |  |  |  |
| Introduction | 1 (1%) | 0 (0%) | 1 (1%) | 0 (0%) | 0 (0%) | 0 (0%) |
| Research question(s) | 6 (4%) | 2 (5%) | 4 (3%) | 3 (3%) | 1 (3%) | 2 (2%) |
| Aim(s) | 21 (13%) | 5 (14%) | 16 (13%) | 20 (17%) | 8 (24%) | 12 (14%) |
| Objective(s) | 72 (45%) | 10 (27%) | 62 (50%) | 39 (33%) | 12 (35%) | 27 (32%) |
| Outcome(s) | 26 (16%) | 9 (24%) | 17 (14%) | 30 (25%) | 6 (18%) | 24 (29%) |
| Outcome measure(s) | 23 (14%) | 7 (19%) | 16 (13%) | 15 (13%) | 4 (12%) | 11 (13%) |
| Analysis | 2 (1%) | 0 (0%) | 2 (2%) | 0 (0%) | 0 (0%) | 0 (0%) |
| Within the text under a feasibility/uncertainty heading | 3 (2%) | 2 (5%) | 1 (1%) | 3 (3%) | 0 (0%) | 3 (4%) |
| Throughout the text, not in one specific area | 6 (4%) | 2 (5%) | 4 (3%) | 8 (7%) | 3 (9%) | 5 (6%) |

Percentages might not add up to 100 due to rounding

*The comparator subset of publications do not report detailed progression criteria but do report prespecified targets relating to recruitment or sample size

**Therapeutic areas that were given in ≥ five publications (reporting progression criteria) are listed; all others are categorised in ‘other’

***Where publications reported a sample size target range (e.g. 12-16 participants), the lower bound of the target is included. A sample size target was not reported in two publications (both reporting completed pilot trials and including the actual number of recruited participants).

****Reported objective is vague (e.g. to ‘assess feasibility’), i.e. specific areas of feasibility uncertainty are not explicitly stated
